# Experimental data for the slug two-phase flow characteristics in horizontal pipeline

**DOI:** 10.1016/j.dib.2017.11.026

**Published:** 2017-11-27

**Authors:** Abdalellah O. Mohmmed, Mohammad S. Nasif, Hussain H. Al-Kayiem

**Affiliations:** a Department of Mechatronics Engineering, Future University, 10553 Africa St, Khartoum, Sudan; b Department of Mechanical Engineering, Universiti Teknologi PETRONAS, 32610 Bandar Seri Iskandar, Perak, Malaysia

## Abstract

The data presented in this article were the basis for the study reported in the research articles entitled “Statistical assessment of experimental observation on the slug body length and slug translational velocity in a horizontal pipe” (Al-Kayiem et al., 2017) [1] which presents an experimental investigation of the slug velocity and slug body length for air-water tow phase flow in horizontal pipe. Here, in this article, the experimental set-up and the major instruments used for obtaining the computed data were explained in details. This data will be presented in the form of tables and videos.

**Specifications Table**

**Table:** 

Subject area	*Fluid Mechanics*
More specific subject area	*Multiphase Flow*
Type of data	*Table, Video*
How data was acquired	*Phantom 9.2 High Speed Camera*
Data format	*analyzed*
Experimental factors	*Before conducting any experimental test, the water tank was ensured to be fully filled and the electrical wires connections were checked. Also, the air pressure in the compressor was ensured to be up to 0.85* *MPa. In addition, the high speed camera was installed in front of the test section after checking the illumination system and capturing pre-video to check the picture quality*
Experimental features	*The experimental tests were conducted in a horizontal Plexiglas transparent test section. The water and air velocities were measured at the inlet of the test section. The measurements of the slug characteristics were performed along two sections located at 58D and 81D from the pipe inlet. The measurements were recorded when the multiphase flow became stabilized at the room temperature of 24 c*^*o*^.
Data source location	*Seri Iskandar, Malaysia*
Data accessibility	*Data is with this article*

**Value of the data**

• A detailed database for the technical slug two-phase flow characteristics in a horizontal 3 in. pipe diameter.
• The significance of this data that it can be used to verify the results of other researcher's models by providing a common benchmark.
• This data can be used for validating the numerical CFD results of the slug characteristics and improving the accuracy of different CFD models in the prediction of slug characteristics.
• The data can be used by other researchers to develop different image processing techniques from the utilized technique in the analysis of this paper. The utilized image process technique is explained in details by (Mohmmed et al. 2016) [2].

## 1 Data

**Fig. 1:**
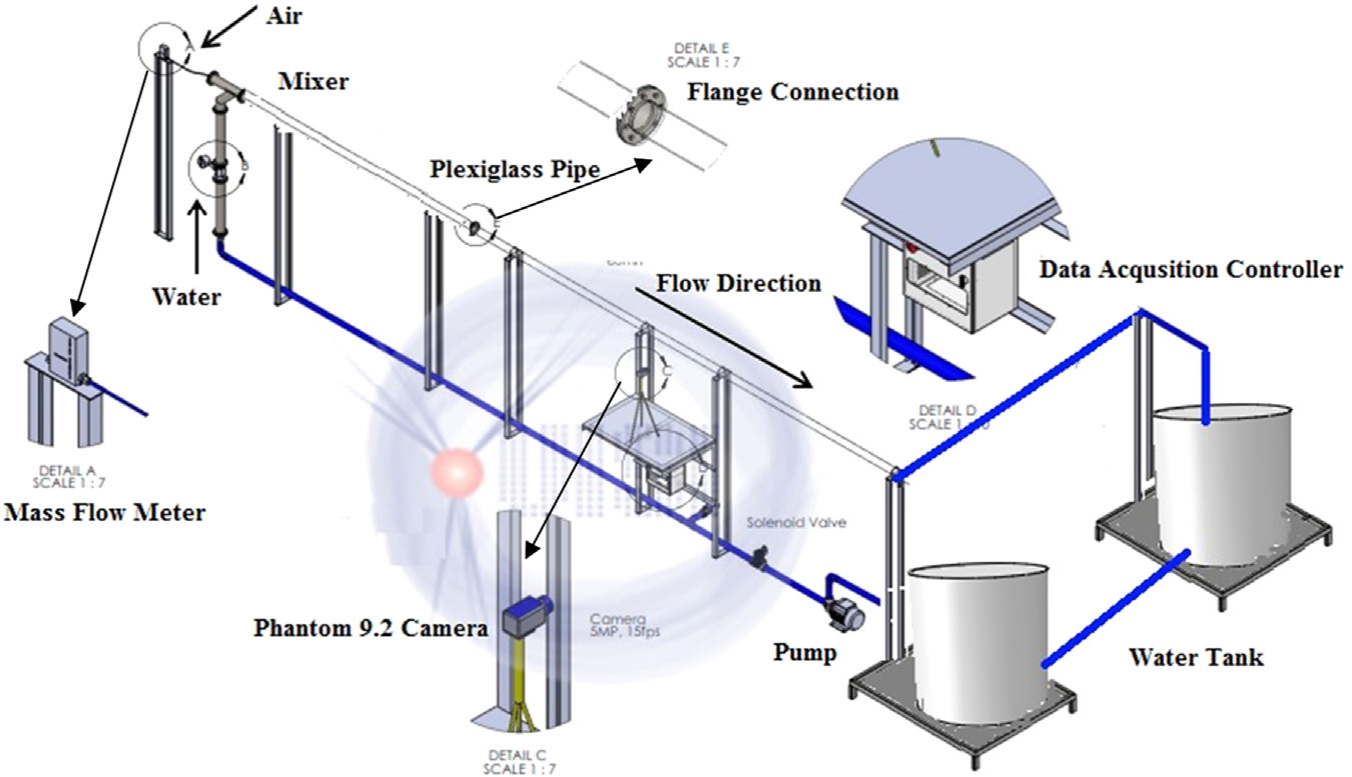
Close loop test rig facilities at Universiti Teknologi PETRONAS Malaysia [1].

The data presented in this article is based on the experimental investigation of the slug two-phase flow in a horizontal circular pipeline (Fig. 1) which was conducted using a close-loop test rig system utilizing the air-water as prime fluids medium [1]. The air-water closed-loop pipeline is used for a wide range of applications [3], [4], [5], [6], [7]. The data used for the investigation of three slug characteristics which are the slug length (Supplementary Table 1), slug frequency (Supplementary Table 2), and the slug translational velocity (Supplementary Table 3) are provided to save time and effort. The data provided was computed from the recorded videos (Supplementary file 4) using a developed Matlab code which explained in (Abdalellah et al.) [2].

## 2 Experimental design, materials and methods

**Fig. 2:**
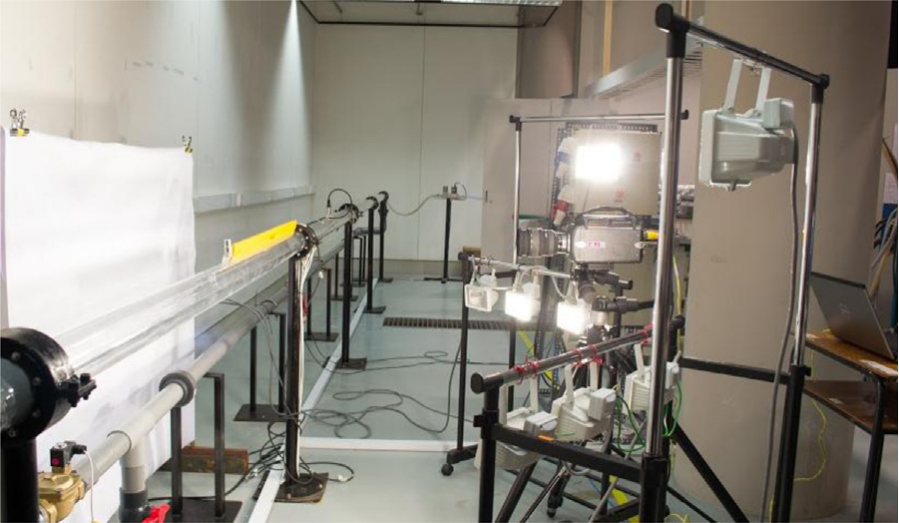
Camera set-up and illumination system.

**Fig. 3:**
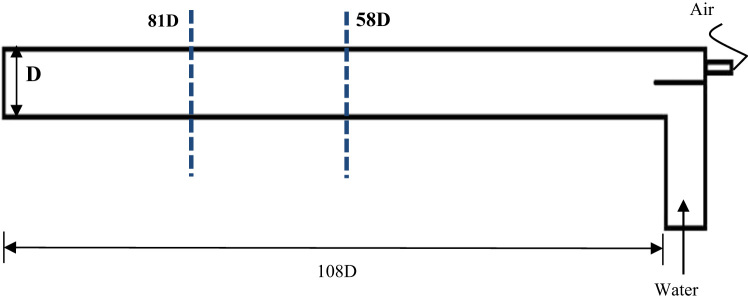
Monitoring sections along the horizontal pipe test section.

The test rig utilized to perform the experimental investigations of air–water slug flow is illustrated in (Fig. 1). A transparent 3 in. diameter pipe with a length of 8 m was fixed on a rigid steel pillars. The water supplied to the test section from two tanks 0.4 and 0.3 m^3^ capacity using a centrifugal (EBARA 3M 50-125/2.2) pump which capable to supply a maximum flow rate of 1 m^3^/min. A solenoid valve was used to control the water supply to the loop. The water and air was separated in the inlet section of the pipe to avoid the perturbation and disturbance. The central compressor was used to supply the air to the test section, which can supply 42.5 m^3^/min of air. The water and air flow were measured using a calibrated ultrasonic flow meter with 70.5% accuracy and a calibrated mass flow controller (Omega FMA-2600A) consecutively. Flow visualization and recording was conducted utilizing a Phantom 9.2 high-speed camera with a recording frequency 1000 frames per second (fps) at the optimum resolution. The recorded videos from the camera was analyzed and processed using a developed in house Matlab code. Fig. 2 illustrates the camera set-up along with the illumination system. In this study, the range of water superficial velocity is from 0.7 m/s to 1.0 m/s, and the range of air superficial velocity from 0.7 m/s to 2.8 m/s. Furthermore, whole experimental investigations were conducted at the room temperature of 24 °C and atmospheric pressure of 1.013 bar. The slug characteristics were monitored at two sections which are 58D and 81D from the pipe inlet section as shown in (Fig. 3).
