# Supplementary material for: Experimental data for the slug two-phase flow characteristics in horizontal pipeline
Source: Data Brief. 2017 Nov 27;16:527–30. doi: 10.1016/j.dib.2017.11.026 (PMC5734701; doi:10.1016/j.dib.2017.11.026)
Supplement: Supplementary file 5 — Supplementary material [file mmc5.docx]

<https://drive.google.com/open?id=0B4R_OZZk58kdT0k3WkplcllJaEU> <https://drive.google.com/open?id=13gXleXaDZVyuxMAE-6VoFq1Se3yDN1Yq>
